# Missed at Birth: A Rare Case of Acute Pancreatitis Secondary to Congenital Diaphragmatic Hernia

**DOI:** 10.1155/2022/7580807

**Published:** 2022-06-16

**Authors:** Ahamed A. Khalyfa, Navkiran Randhawa, David Gabbert, Ashirf Al-Ghanoudi

**Affiliations:** Franciscan Health Olympia Fields, Department of Gastroenterology, Olympia Fields, IL, USA

## Abstract

Acute pancreatitis is a common gastrointestinal cause of hospitalizations across the world. The most common etiologies of acute pancreatitis include gallstones, excessive alcohol use, hypertriglyceridemia, or, rarely, trauma. Traction-induced pancreatitis is an uncommon but previously reported cause of acute pancreatitis. We present a 60-year-old male with a past medical history of cerebral palsy who presented to our facility with acute pancreatitis secondary to a congenital diaphragmatic hernia.

## 1. Introduction

Acute pancreatitis is an inflammatory condition of the pancreas defined by at least two of the following: abdominal pain, elevated serum lipase levels at least three times the normal level, and/or characteristic findings on imaging. The annual incidence rate of acute pancreatitis ranges from 4.9 to 35 per 100,000 patients in the United States making it one of the most common gastroenterological emergencies [1]. The three main etiological factors attributing to acute pancreatitis in up to 70%, 35%, and 14% of cases are gallstones, alcohol use, and hyperlipidemia, respectively [2–4]. Diaphragmatic hernia-induced acute pancreatitis is a rare entity.

Diaphragmatic hernias are defined by herniation of the abdominal viscera into the thoracic cavity. Typically, diaphragmatic hernias are congenital or acquired in adulthood due to blunt or penetrating injury [5]. Congenital diaphragmatic hernias (CDHs) occur due to abnormal development of the diaphragm [5]. CDH commonly is diagnosed at birth as it presents as a life-threatening breathing difficulty. Rarely, these hernias are asymptomatic at birth and cause complications later on in life. Bochdalek hernias are considered the most common type of CDH [6]. Eighty-five percent of these posterior lateral hernias occur on the left side, and thirteen percent occur on the right [6].

To our knowledge, this is the first case of acute pancreatitis secondary to a congenital diaphragmatic hernia in an adult above the age of 60.

## 2. Case Report

**Figure 1:**
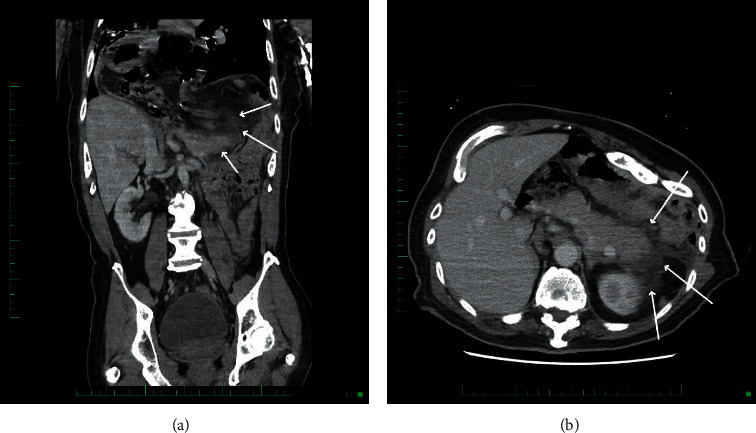
Coronal image (a) showing the peripancreatic inflammation (white arrows) in the LUQ around the tail of the pancreas. Axial image (b) showing the inflammation with the edematous appearance of the pancreatic body and tail (the brighter structure that the arrows are pointing to).

The patient is a 60-year-old male with a past medical history of cerebral palsy and congenital diaphragmatic hernia who presented with intractable vomiting. The patient's symptoms started 1 day prior to admission and his vomitus was nonbloody and nonbiliary. The patient also complained of mild abdominal discomfort but otherwise had no complaints. Nasogastric tube was inserted and programmed to low intermittent suction. Upon presentation, the patient was hemodynamically stable and laboratory findings were significant for lipase >3000 (ref 11–82 U/L). Alkaline phosphatase was 13 and T Bili was 0.3. CT abdomen and pelvis w*w* contrast showed pancreatic edema and moderate free fluid most pronounced at the pancreatic tail which was consistent with pancreatitis (Figure 1). There was no evidence of pancreatic duct dilatation at the head or body of the pancreas.

Furthermore, a large diaphragmatic hernia with herniation of the entirety of the stomach and portion of colon into the right thorax with no evidence of bowel strangulation was also demonstrated on CT (Figure 1). There was no evidence of hepatobiliary or pancreatic masses on imaging. Given that the patient had no prior alcohol history, a gallbladder dedicated ultrasound was performed which showed no evidence of common bile duct dilation, gallstones, gallbladder wall thickening, pericholecystic fluid, or choledocholithiasis. The patient's symptoms subsided within one day and lipase downtrended to 200 U/L with supportive care. Given the patient's lack of alcohol history, lack of gallstones, no evidence of malignancy, and rapid resolution of symptoms, the patient's pancreatitis was deemed to be most likely secondary to severe diaphragmatic hernia. Due to the patient's functional status with his cerebral palsy, a shared decision was made to not pursue invasive surgical treatments and to continue conservative measures.

## 3. Discussion

Acute pancreatitis is the leading cause of gastrointestinal-related hospitalizations in the United States, and its prevalence is on the rise both nationally and internationally [7]. In the majority of cases, acute pancreatitis is caused by excessive alcohol intake, gallstones, and hypertriglyceridemia. The pathophysiology of pancreatitis incorporates both the localized destruction of the pancreas and an inflammatory response [8]. It often includes elevation in ductal pressures which can be caused by compression or obstruction of the pancreatic ducts as well as problems with calcium homeostasis [8].

The majority of cases of acute pancreatitis in adults secondary to hernia have been associated with hiatal hernias or traumatic diaphragmatic hernias. Acute pancreatitis secondary to a diaphragmatic hernia is an extremely rare phenomenon. Adult-onset pancreatitis secondary to a congenital diaphragmatic hernia is almost unheard of, with only 2 prior cases reported in the literature, one patient 27 years of age and the other 35 years of age [9, 10]. To our knowledge, this is the first reported case in the literature of pancreatitis secondary to a congenital diaphragmatic hernia in an adult greater than 60 years of age.

There is no clear pathophysiologic etiology for pancreatitis caused by diaphragmatic hernia; however, several purported mechanisms exist including abnormal pancreatic traction causing pancreatic duct drainage obstruction, incarceration of the pancreas within the hernia without volvulus, repetitive trauma through crossing of the hernial sac, and traction ischemia of the pancreatic vascular supply [11].

Of the cases previously reported regarding acute pancreatitis in the setting of diaphragmatic hernia, all of them required emergent surgical intervention due to incarceration or volvulus. Our case is unique as our patient was treated conservatively with considerable improvement in his lipase level and symptomatology. In our case, the patient most likely had intermittent blockage of blood supply and/or pancreatic duct flow which spontaneously resolved.

## 4. Conclusion

In conclusion, we report the first known case of acute pancreatitis secondary to a congenital diaphragmatic hernia in an adult above the age of 60. Moreover, although lipase is commonly not recommended to be trended for pancreatitis, this case shows some evidence that lipase trend may provide some clinical benefit in the specific setting of pancreatitis secondary to a diaphragmatic hernia. Further studies will be necessary to confirm this potential clinical association.

## Data Availability

No data were used to support this study.
